# Relationships between Breastfeeding Patterns and Maternal and Infant Body Composition over the First 12 Months of Lactation

**DOI:** 10.3390/nu10010045

**Published:** 2018-01-05

**Authors:** Zoya Gridneva, Alethea Rea, Anna R. Hepworth, Leigh C. Ward, Ching T. Lai, Peter E. Hartmann, Donna T. Geddes

**Affiliations:** 1 School of Molecular Sciences, M310, The University of Western Australia, Crawley, Perth, WA 6009, Australia; arhepworth@westnet.com.au (A.R.H.); ching-tat.lai@uwa.edu.au (C.T.L.); peter.hartmann@uwa.edu.au (P.E.H.); donna.geddes@uwa.edu.au (D.T.G.); 2 Centre for Applied Statistics, The University of Western Australia, Crawley, Perth, WA 6009, Australia; alethea.rea@uwa.edu.au; 3 School of Chemistry and Molecular Biosciences, The University of Queensland, St. Lucia, Brisbane, QLD 4072, Australia; l.ward@uq.edu.au

**Keywords:** human milk, breastfed infants, body composition, anthropometrics, milk intake, bioelectrical impedance spectroscopy, ultrasound skinfolds, maternal factors

## Abstract

Breastfeeding has been implicated in the establishment of infant appetite regulation, feeding patterns and body composition (BC). A holistic approach is required to elucidate relationships between infant and maternal BC and contributing factors, such as breastfeeding parameters. Associations between maternal and breastfed term infant BC (*n* = 20) and feeding parameters during first 12 months of lactation were investigated. BC was measured at 2, 5, 9 and/or 12 months postpartum with ultrasound skinfolds (US; infants only) and bioimpedance spectroscopy (infants and mothers). 24-h milk intake (MI) and feeding frequency (FFQ) were measured. Higher FFQ was associated with larger 24-h MI (*p* ≤ 0.003). Higher 24-h MI was associated with larger infant fat mass (FM) (US: *p* ≤ 0.002), greater percentage FM (US: *p* ≤ 0.008), greater FM index (FMI) (US: *p* ≤ 0.001) and lower fat-free mass index (FFMI) (US: *p =* 0.015). Lower FFQ was associated with both larger FFM (US: *p* ≤ 0.001) and FFMI (US: *p* < 0.001). Greater maternal adiposity was associated with smaller infant FFM measured with US (BMI: *p* < 0.010; %FM: *p* = 0.004; FMI: *p* < 0.011). Maternal BC was not associated with FFQ or 24-h MI. These results reinforce that early life is a critical window for infant programming and that breastfeeding may influence risk of later disease via modulation of BC.

## 1. Introduction

The importance of lactocrine programming has been highlighted recently, with breastfeeding identified as one of the most economical preventative measures for non-communicable diseases (NCD) including obesity later in life [1,2,3,4,5]. The development of body composition (BC) in early life is known to play an important role in the programming of these health outcomes [6]. This reduction in risk may be a result of multiple mechanisms associated not only with composition of human milk (HM) but also with infant breastfeeding patterns and behavior [7,8,9], all of which may influence the growth and development of breastfed infants. Differences in the weight and BC between breastfed and formula-fed infants have been attributed to the stark compositional differences of HM and formula [10,11]. Despite the evidence that volume of HM is a main driver of growth [11,12,13], a major focus of at present limited research on infant growth and BC development has been on the composition of HM and maternal pre-pregnancy body mass index (BMI), and to a lesser extent on the effect of the volume of HM and maternal adiposity. Although these findings suggest a dose-dependent effect of breastfeeding on development of infant BC, the pathways of this effect are not fully understood.

Indeed, in utero maternal influences are apparent in obese women who generally deliver heavier infants with greater adiposity [14] thus maternal weight is a major predictor of infant birth weight (BW) [15,16]. However, recent studies have shown the infant BW is not associated with increased maternal BMI in women with BMI above 24 kg/m^2^ [17]. To further complicate our understanding, overweight women deliver infants with higher adiposity [18] but not fat-free mass (FFM) [19,20]. Unfortunately, the majority of the studies have analyzed maternal pre-pregnancy BMI or gestational weight gain (parameters that are often self-reported and potentially misleading) as measures of adiposity. Considering that HM composition is influenced by the current maternal BC [21] rather than pre-pregnancy BMI, longitudinal studies with multiple measures of both maternal and breastfed infant BC are necessary [22] to elucidate the positive mechanistic effects of breastfeeding. 

**Figure 1:**
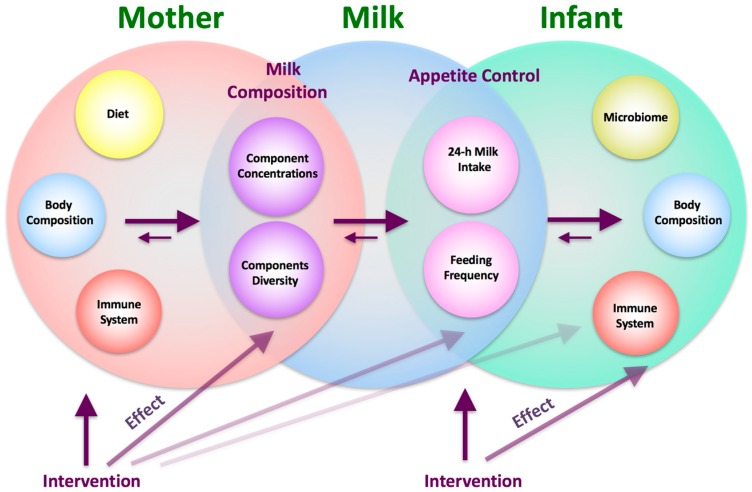
Framework for possible interconnecting pathways of lactocrine programming of the infant, and points of intervention for potential improvement of infant growth, development and health, based on available research.

In a few recent studies, maternal BC was measured during pregnancy and a positive association between maternal BC and infant BW was found [23,24,25,26], showing maternal FFM or total body water, but not fat mass (FM) are the strongest predictors. Interestingly, longer duration of breastfeeding is shown to attenuate the adverse effects of BW and early weight gain on infant FM gain [27]. Further, the majority of participants are either newborns or children between 2 and 11 years of age, and infant adiposity (fat mass (FM) and percentage FM (%FM)) has been measured rather than FFM, yet the metabolic rate is largely determined by the FFM [28,29]. Furthermore, despite 24-h milk intake (MI) having a strong positive relationship with infant weight gain [12,13,30], there has been no investigation of the effect of either 24-h MI or feeding frequency (FFQ) on infant BC, yet these factors are highly variable between infants [31]. Our recent research of gastric emptying in term breastfed infants indicated that shorter, smaller and leaner infants fed more frequently (maternal self-report) [32], highlighting the need to connect not only maternal and infant BC, but also contributing factors, such as milk production and composition, infant MI and FFQ and, in turn, the development of the breastfed infant BC (Figure 1).

It is important to understand the mechanisms by which maternal BC, breastfeeding and HM components may influence infant BC, as this will allow for more targeted interventions that may potentially reduce both infant and adult overweight and obesity. Therefore, the aim of this longitudinal study was to investigate relationships between maternal and infant BC during the first 12 months of lactation. Further, exploration of relationships of infant 24-h MI and FFQ with maternal and infant BC was carried out.

## 2. Materials and Methods

### 2.1. Study Participants

Breastfed infants (*n* = 20; 10 males, 10 females) of English-speaking, predominantly Caucasian, mothers of higher social-economic status from a developed country were recruited from the community, primarily from the West Australian branch of the Australian Breastfeeding Association. Inclusion criteria were: healthy singletons, gestational age ≥37 weeks, exclusively breastfed [33] at 2 and 5 months, and maternal intention to breastfeed until 12 months. Exclusion criteria were: infant factors that could potentially influence growth and development of BC, maternal smoking, and low milk supply. All mothers provided written informed consent to participate in the study, which was approved by The University of Western Australia Human Research Ethics Committee (RA/1/4253, RA/4/1/2639) and registered with the Australian New Zealand Clinical Trials Registry (ACTRN12616000368437).

### 2.2. Study Session

Measurements were made when the infants were 2 and/or 5, 9 and 12 months of age. Participants visited our laboratory at King Edward Memorial Hospital for Women (Subiaco, Perth, WA, Australia) for up to four monitored breastfeeding sessions between March 2013 and September 2015. At each study session, the infant was weighed pre-feed, and then the mother breastfed her infant. Infant bioelectrical impedance spectroscopy (BIS) measurements were made pre-feed, unless impractical, then they were made post-feed [34]. Ultrasound skinfold (US) and anthropometric measurements were made post-feed. This combination of methods for measuring infant BC was used to ensure safe, non-invasive and accurate assessment, and to avoid the inherent limitations of a singular technique [35]. Clothing was removed for the measurements except for a dry diaper and a singlet.

Maternal weight, height and BIS measurements were recorded. Current FFQ of the infants was self-reported by mothers.

### 2.3. Anthropometric Measurements

Infants weight was determined before breastfeeding using Medela Electronic Baby Weigh Scales (±2.0 g; Medela Inc., McHenry, IL, USA). Infant crown-heel length was measured once to the nearest 0.1 cm using non-stretch tape and a headpiece and a footpiece, both applied perpendicularly to a hard surface. Infant head circumference was measured with a non-stretch tape to the nearest 0.1 cm.

Maternal weight was measured using an electronic scale (±0.1 kg; Seca, Chino, CA, USA). Height was self-reported by participants or measured against a calibrated marked wall (accuracy ± 0.1 cm).

Infant and maternal BMI were calculated as kg/m^2^. 

### 2.4. Body Composition with Bioelectrical Impedance Spectroscopy

Whole body bioimpedance (wrist to ankle) of infants and mothers was measured using the Impedimed SFB7 bioelectrical impedance analyzer (ImpediMed, Brisbane, QLD, Australia) according to the manufacturer’s instructions. 

Mothers were measured in supine position on a non-conductive surface. A series of ten consecutive measurements (fat mass (FM), percentage fat mass (%FM) and fat-free mass (FFM)) were taken within 1–2 min and averaged for data analysis. The within participant coefficient of variation (CV) for maternal %FM was 0.21% [21].

Infants were measured by applying an adult protocol as used previously in infants but with data analyzed using settings customized for infants [35,36]. Resistance (ohm) at 50 kHz (R_50_) was determined from the curve of best fit, averaged for analysis purposes and used in the Lingwood et al. [36] age-matched (3 and 4.5 month-old infants) BIS equations for FFM of 2 and 5 month-old infants respectively, and Bocage [37] total body water (TBW) equations for 9 and 12 month-old infants:

TBW = (0.418 × Weight (kg) + 1936/R_50_ + 0.8649) × Length (cm)/100 (1)

FFM was further determined using sex and age-appropriate hydration factors (HF) calculated from Butte et al. [38]:

FFM = TBW/HF.(2)

%FM was calculated as follows:

%FM = 100 × (Weight (kg) − FFM (kg))/Weight (kg).(3)

Within participant CV for infant R_50_ was 1.5% [34].

### 2.5. Ultrasound Skinfold Measurements

Infant skinfolds were measured using the Aplio XG (Toshiba, Tokyo, Japan) US machine with a 14–8 MHz transducer (PLT-1204BX) and sterile water-based ultrasonic gel (Parker Laboratories Inc., Fairfield, NJ, USA) as described previously [35]. Single US scans of four anatomical sites (biceps, subscapular, suprailiac and triceps) were performed on the left side of the body with minimal compression. Subcutaneous tissue thickness (skin thickness and the skin-fat interface to fat-muscle interface distance) was measured directly from images on the screen using electronic calipers. One experienced sonographer with good interrater reliability [39] performed all of the measurements. US measurements were doubled [40] for use in skinfold equations developed for subcutaneous tissue thickness measurement with skinfold calipers. At all time points, infant %FM with 2-skinfolds (US 2SF: triceps, subscapular; Slaughter et al. [41]) and density (*d*; kg/L) with 4-skinfolds (US 4SF: biceps, subscapular, suprailiac and triceps; Brook [42]) were calculated with %FM further determined using Lohman equation [43]:

%FM = 100 × (5.28/*d* − 4.89). (4)

### 2.6. Body Composition Indices

The indices of height-normalized BC were calculated for mothers and infants: fat mass index (FMI) was calculated as FM/length^2^, and fat-free mass index (FFMI) was calculated as FFM/length^2^; both expressed as kg/m^2^ [44].

### 2.7. 24-H Milk Intake and Feeding Frequency

Infant MI was measured by mothers using the 24-h milk production (MP) protocol, weighing infants at home with the Medela Electronic Baby Weigh Scales pre- and post each breastfeed during a 24-h period plus one breastfeeding, and recording amounts of HM (g) consumed by the infant (including expressed HM if any) [45]. 24-h MI was determined as previously described with potential underestimation of 3–10% [45] and FFQ (meals per day) was recorded [31]. 24-h MI was measured at three time points: between 2 and 5 (4.0 ± 1.3) months, when MI is shown to be stable [31], and within two weeks of 9 (9.4 ± 0.3) and 12 (12.2 ± 0.4) months. Given that measuring 24-h MI is not always practical, particularly at the later stages of lactation, mothers were also asked how frequently the infant fed, and self-reported the typical time between the feeds (e.g., each 2 h) during the week prior to the study session as a proxy measure of FFQ.

### 2.8. Statistical Analyses

Statistical analysis was performed in R 3.1.2 for Mac OSX [46]. Additional packages were used for linear mixed effects models (nlme, lme4 and car) [47,48,49], intra-class correlations (irr) [50], Tukey’s all pair comparisons (multcomp) [51] and graphics (ggplot2) [52]. Descriptive statistics are reported as mean ± standard deviation (SD) (range); model parameters as estimate ± SE (standard error).

During this longitudinal study infants were measured at four time points (2 and/or 5, 9 and 12 months). An approximate sample size was calculated using the ‘F tests–Linear multiple regression: Fixed model: *R*^2^ increase’ option in G*Power [53] as if this was a cross-sectional study with equal numbers at each time. Allowing four predictors (3 for age comparisons), α = 0.05 and 14 participants (56 sample points = 14 participants × 4 time points) gave the study power of 0.80 to detect an effect size of 0.15. This approach was selected, as there is no closed form expression suitable for the calculation of sample sizes for this research design [54], with the consideration that longitudinal study design is more powerful. Recruitment of participants at the 5 months point was introduced, as many mothers would not commit to a study that required breastfeeding to 12 months, when approached at 2 months (*n* = 8). As a result, required number of participants was increased to 20 in order to maintain predicted power; this also addressed issues relating to missed visits. Missing data was dealt with using available case analysis.

Maternal BC was analysed using an intercept only linear mixed effects model for the calculation of CV for maternal %FM measurements (*n* = 10, 10 measurements each).

Infant BC was analysed using linear mixed effects models with random intercept per participant to determine whether BC measurements (%FM, FM, FMI, FFM and FFMI) differed systematically by age, measurement method (US 2SF, US 4SF and BIS) and infant sex. As interactions between sex and methods were non-significant (*p* > 0.52), reported associations are for pooled data. Months after birth were accounted for in all models; results reported account for this, regardless of significance.

Survey responses relating to FFQ were analysed using a one-way intra-class correlation for agreement of single measures.

The analyses for systematic differences in all measured parameters (maternal characteristics, infant characteristics and breastfeeding characteristics) at different months after birth and between different measurement methods used general linear hypothesis tests (Tukey’s all pair comparisons).

Relationships between infant BW and maternal and infant BC at four time points after birth were analysed using linear regression models accounting for gestational age and sex, which were identified as significant covariates using a stepwise regression analysis. Since major postpartum weight/adiposity loss happens during the first 4 to 6 months in women of high social-economic status [55,56], maternal BC at 5, 9 and 12 months was considered instead of unavailable pre-pregnancy BMI.

Relationships between: (a) infant BC and breastfeeding characteristics; (b) breastfeeding characteristics and maternal BC; and (c) infant and maternal BC were analysed using linear mixed effects models. Each breastfeeding characteristic or infant BC measure/index was considered separately as the response variable, and each model contained fixed effects of infant age (months), a predictor (breastfeeding measure or maternal BC measure/index) and an interaction between infant age and predictor, as well as a random intercept per participant.

Owing to the large number of comparisons, a false discovery rate adjustment [57] was performed on associated subgroupings of results. *p*-values were considered to be significant below 0.018 for associations between infant BW and maternal BC; below 0.047 for associations between infant BW and infant BC; below 0.018 for associations between infant FFM and maternal BC; below 0.038 for associations between infant FFMI and maternal BC; below 0.029 for associations between infant BC and MI; below 0.040 for associations between infant BC and self-reported FFQ; below 0.05 for associations between infant BC and 24-h MP FFQ; below 0.0004 for associations between infant BC changes between the time points and 24-h MI; below 0.001 for associations between infant BC changes between the time points and self-reported FFQ; below 0.004 for associations between infant BC changes between the time points and 24-h MP FFQ; below 0.009 for associations between maternal BC changes between time points and 24-h MI; below 0.014 for associations between maternal BC changes between time points and self-reported FFQ; and below 0.05 for associations between maternal BC changes between time points and 24-h MP FFQ. The significance was set at the 5% level otherwise.

## 3. Results

### 3.1. Subjects

Twenty-two infants were recruited; 2 infants (1 male, 1 female) were excluded from the study after the 2-month visit (starting supplementation with formula; personal circumstances). One female infant started supplementation with formula/weaning at 6 months and was excluded from further analysis. Nineteen remaining infants were breastfed at 2, 5 and 9 months. Seventeen infants (94%) continued to breastfeed at 12 months, but one male was too sick to attend the last session. Out of 18 infants measured at 12 months 16 infants (89%) still continued to breastfeed. One male infant ceased breastfeeding 2 weeks before the 12-month appointment and one female infant stopped at 10 months after birth. Both infants and their mothers were measured at 12 months. 

Some sessions were not attended by some participants leading to incomplete data. Five infants did not start at 2 months, two did not attend at 9 months and two at 12 months. Overall 80 measures were expected, however some were missing, specifically: infant weight (*n* = 9); infant %FM, FM, FMI, FFM and FFMI measured with US 2SF, and maternal age, weight, height, BMI, %FM, FM, FMI, FFM and FFMI (*n* = 10); infant head circumference (*n* = 11); infant length, BMI and %FM, FM, FMI, FFM and FFMI measured with US 4SF (*n* = 12); infant %FM, FM, FMI, FFM and FFMI measured with BIS (*n* = 13); self-reported FFQ (*n* = 20). Missing data also occurred due to difficulties with conducting 24-h MI measurements at later stages of lactation. The following measurements from the 60 expected were missing: FFQ from 24-h MP (*n* = 26) and 24-h MI (*n* = 27). Missing data were spread across the time points (Table 1).

Breastfeeding characteristics, infant and maternal demographics and anthropometrics as well as maternal BC measures at the four study sessions are presented in Table 1. Mean maternal age at the start of the study was 33.3 ± 4.7 (24–44) years, mean height was 167.4 ± 7.4 (150–181) cm and mean parity was 2.3 ± 0.9 (1–4). Infant male/female ratio was 10/10, mean BW was 3.486 ± 0.498 (2.660–4.455) kg and mean gestational age was 39.4 (37.6–43) weeks. After accounting for infant age males were heavier (0.85 [0.12, 1.57], *p* = 0.025) and had larger head circumferences than females (1.89 [0.81, 2.96], *p* = 0.002), while no significant difference between sexes was seen for either length (1.68 [−0.24, 3.59], *p* = 0.083) or BMI (1.09 [−0.15, 2.32], *p* = 0.081). 

### 3.2. Maternal Body Composition

Maternal BC is presented in Table 1. At the session attended at 5 months postpartum none of the participants were classified as being underweight (BMI < 18.5; %FM < 21). They were classified as: normal weight (BMI 18.5–24.9, 65%, *n* = 13; %FM 21–32.9, 55%, *n* = 11), overweight (BMI 25–29.9, 20%, *n* = 4; %FM 33–38.9, 30%, *n* = 6) or obese (BMI > 30, 15%, *n* = 3; %FM > 39, 15%, *n* = 3) [58].

### 3.3. Infant Body Composition

Infant BC measured with three measurement techniques (BIS, US 2SF and US 4SF) is presented in Table S1. 

Male infants were compared to female infants using all three measurement techniques. FFM was significantly greater in males overall (0.66 [0.19, 1.14] kg, *p* = 0.009) and when the methods were considered separately (US 2SF: 0.55 [0.07, 1.03] kg, *p* = 0.027; US 4SF: 0.70 [0.20, 1.20] kg, *p* = 0.009; BIS: 0.74 [0.25, 1.22] kg, *p* = 0.005). FFMI was significantly higher in males overall (0.95 [0.21, 1.69] kg, *p* = 0.015) and when determined with US 4SF and BIS (US 4SF: 1.01 [0.29, 1.73] kg, *p* = 0.009; BIS: 1.09 [0.37, 1.81] kg, *p* = 0.005) but not with US 2SF (0.70 [−0.12, 1.52] kg, *p* = 0.089). 

Differences were not seen for %FM, FM and FMI overall (males %FM: −0.38 [−3.02, 2.26] %, *p* = 0.77; FM: 0.19 [−0.17, 0.55] kg, *p* = 0.27; FMI: 0.16 [−0.52, 0.85] kg/m^2^, *p* = 0.62) or when the methods were considered separately (%FM: *p* ≥ 0.30; FM: *p* ≥ 0.095; FMI: *p* ≥ 0.25). 

A comparison of measurement methods showed no difference for %FM (*p* ≥ 0.074), FM (*p* ≥ 0.11), FMI (*p* ≥ 0.077) and FFM (*p* ≥ 0.15). Overall FFMI determined with BIS was significantly higher compared with US 2SF (0.24 ± 0.10, *p* > 0.039) with no further differences between the methods (*p* ≥ 0.24).

### 3.4. Infant Birth Weight and Maternal and Infant Body Composition

After accounting for infant sex and gestational age no significant associations between BW and any maternal BC parameter were seen at any time point after birth after adjusting for the false discovery rate (*p* ≥ 0.018) (the raw *p*-values for negative associations between maternal adiposity/BC indices at 5, 9 and 12 months postpartum and infant BW before the adjustment were: %FM (5 months: −0.03 ± 0.01, *p* = 0.026; 9 months: −0.03 ± 0.01, *p* = 0.021; 12 months: −0.03 ± 0.01, *p* = 0.018), BMI (5 months: −0.04 ± 0.02, *p* = 0.024; 9 months: −0.03 ± 0.02, *p* = 0.046; 12 months: −0.04 ± 0.02, *p* = 0.019), FMI (5 months: −0.06 ± 0.02, *p* = 0.032; 9 months: −0.05 ± 0.02, *p* = 0.038; 12 months: −0.06 ± 0.02, *p* = 0.023), and FFMI (5 months: −0.10 ± 0.04, *p* = 0.042; 12 months: −0.09 ± 0.04, *p* = 0.032)).

After accounting for infant sex and gestational age and adjusting for the false discovery rate (*p* ≥ 0.047) larger BW was associated with larger infant FFM measured at all-time points and with all three methods (2 months: US 2SF, 0.81 ± 0.17, *p* = 0.001; US 4SF, 0.95 ± 0.23, *p* = 0.004; BIS, 0.75 ± 0.22, *p* = 0.010; 5 months: US 2SF, 1.03 ± 0.30, *p* = 0.004; US 4SF, 1.24 ± 0.29, *p* < 0.001; BIS, 0.87 ± 0.17, *p* < 0.001; 9 months: US 2SF, 1.20 ± 0.32, *p* = 0.002; US 4SF, 1.17 ± 0.33, *p* = 0.004; BIS, 1.37 ± 0.39, *p* = 0.004; 12 months: US 2SF, 1.46 ± 0.32, *p* < 0.001; US 4SF, 1.55 ± 0.32, *p* < 0.001; BIS, 1.42 ± 0.42, *p* = 0.006). Also, larger BW was associated with larger FM only at 5 months and only when measured with BIS (0.70 ± 0.25, *p* = 0.014).

### 3.5. 24-H Milk Intake and Feeding Frequency

A moderate level of agreement (ICC = 0.602 [0.339, 0.779], *p* < 0.001) was seen between FFQ measured with 24-h MP as meals per 24-h and FFQ self-reported by mothers as hours between meals. Short intervals between feeds were associated with higher self-reported values than 24-h MP values; this effect was not seen with longer intervals between feeds. 

FFQ and 24-h MI did not differ by infant sex (*p* ≥ 0.54). Greater FFQ was associated with larger 24-h MI (24-h MP FFQ: 81.1 ± 18.5, *p* < 0.001; self-reported FFQ: −50.6 ± 13.3, *p* = 0.003). 

### 3.6. Longitudinal Changes in Maternal, Breastfeeding and Infant Characteristics

**Figure 2:**
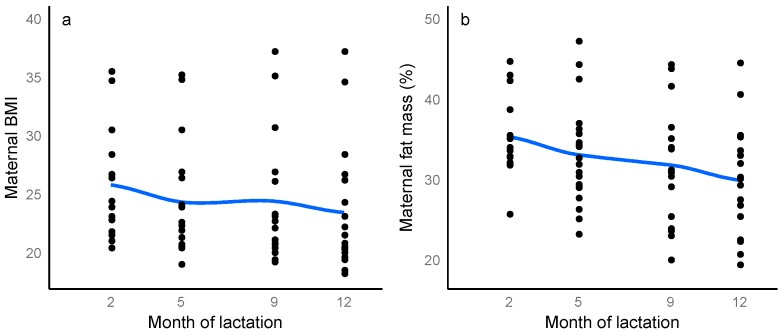

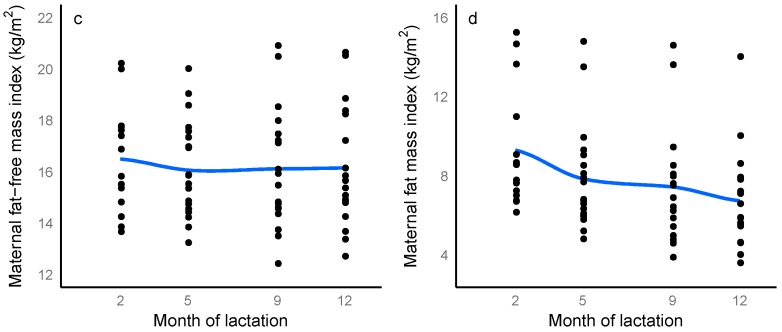
Longitudinal changes in: (**a**) maternal body mass index (BMI); (**b**) maternal percentage fat mass, (**c**) fat-free mass index and (**d**) fat mass index measured with bioelectrical impedance spectroscopy from 2 to 12 months of lactation. Blue line represents local regression smoother (LOESS), grey areas represent ± confidence interval, (*n* = 14, *n* = 20, *n* = 18, *n* = 18 at 2, 5, 9 and 12 months respectively).

Maternal weight, BMI, %FM, FM and FMI decreased significantly between 2 and 12 months (Table S2), while FFM and FFMI did not differ (FFM: *p* = 0.10; FFMI: *p* = 0.076). Over the first year of lactation, maternal adiposity decreased (Figure 2) (%FM: −2.03% ± 0.59, *p* = 0.001, month of lactation: *p* < 0.001; BMI: −0.78 ± 0.24, *p* = 0.002, month of lactation: *p* < 0.001; FMI: −0.64 ± 0.18, *p* < 0.001, month of lactation: *p* = 0.001), after accounting for the month of lactation as a factor.

FFQ and 24-h MI decreased significantly across the lactation (Table S2).

**Figure 3:**
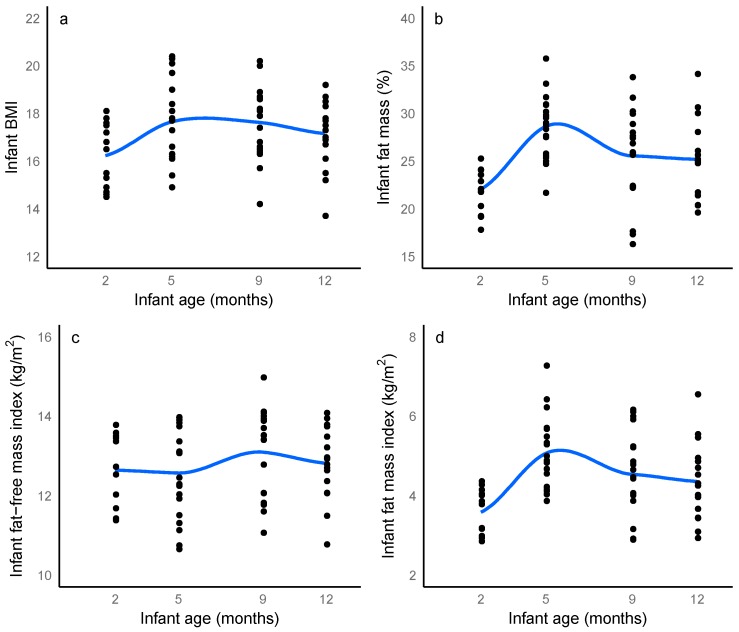
Longitudinal changes in: (**a**) infant body mass index (BMI); (**b**) percentage fat mass, (**c**) infant fat-free mass index and (**d**) infant fat mass index from 2 to 12 months after birth measured with bioelectrical impedance spectroscopy. Blue line represents local regression smoother (LOESS), grey areas represent ± confidence interval, (*n* = 14, *n* = 20, *n* = 18, *n* = 18 at 2, 5, 9 and 12 months respectively).

Infant anthropometrics and both FM and FFM measured with all methods increased significantly as age increased (Table S3). BMI, FFMI determined with US 4SF, FMI determined with US 2SF and FM determined with BIS initially increased and then plateaued, while %FM and FMI measured with BIS initially increased and then decreased (Figure 3). %FM measured with US skinfolds and FFMI determined with US 2 SF did not differ significantly (%FM US 2SF: *p* = 0.56; %FM US 4 SF: *p* = 0.11; FFMI US 2SF: *p* = 0.13).

### 3.7. Relationships between Infant and Maternal Body Composition

**Figure 4:**
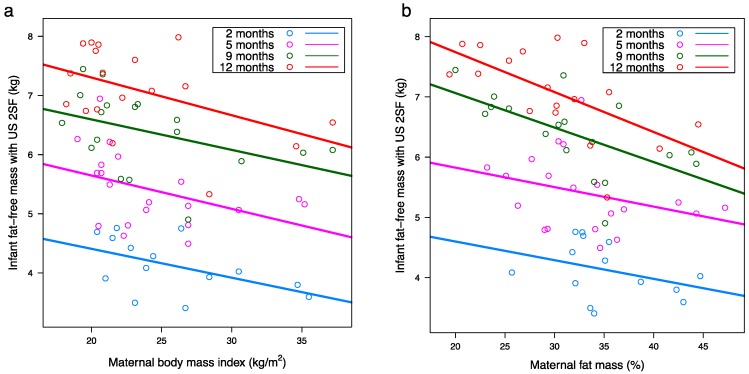
Significant negative associations between: (**a**) maternal body mass index and infant fat-free mass measured with ultrasound (2 skinfolds; US 2SF); (**b**) maternal percentage fat mass (%FM) and infant fat-free mass measured with US 2SF. Lines represent linear regression, one line for each time point (*n* = 14, *n* = 20, *n* = 18, *n* = 18 at 2, 5, 9 and 12 months respectively).

Significant negative associations between infant FFM and maternal adiposity were seen after accounting for month after birth and interaction between month after birth and maternal characteristic (Table A1). After adjusting for the false discovery rate, higher maternal BMI was associated with smaller infant FFM measured with both US 2SF (*p* = 0.007) and US 4SF (*p* = 0.010) (Figure 4a); greater maternal FM was associated with smaller infant FFM measured with US 2SF (*p* = 0.004) (Figure 4b); and greater maternal FMI was associated with smaller infant FFM measured with both US 2SF (*p* = 0.005) and US 4SF (*p* = 0.011). There were no other significant associations between the measured maternal and infant BC parameters. No significant interactions between month after birth and maternal predictors were seen.

### 3.8. Infant Body Composition and Breastfeeding Parameters

Significant associations between infant BC and feeding parameters (FFQ, 24-h MI) were seen after accounting for the month after birth and interaction between month after birth and feeding parameters (Table A2). 

**Figure 5:**
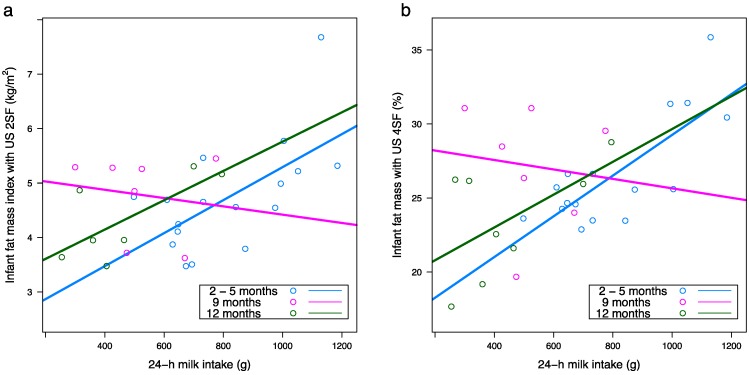
Significant positive associations between: (**a**) 24-h milk intake and infant fat mass index measured with ultrasound (2 skinfolds; US 2SF) (*n* = 17, *n* = 7, *n* = 7 between 2 and 5, and at 9 and 12 months respectively); (**b**) 24-h milk intake and infant percentage fat mass (%FM) measured with ultrasound (4 skinfolds; US 4SF) (*n* = 16, *n* = 7, *n* = 7 between 2 and 5, and at 9 and 12 months respectively). Lines represent linear regression, one line for each time point.

After adjusting for false discovery rate, higher 24-h MI was associated with greater infant FM measured with both US 2SF (*p* = 0.004) and US 4SF (*p* = 0.002), greater %FM measured with both US 2SF (*p* = 0.008) and US 4SF (*p* < 0.001), greater FMI measured with US 2SF (*p* = 0.001) and US 4SF (*p* < 0.001) and lower FFMI measured with US 4SF (*p* = 0.015) (Table A2, Figure 5). 

**Figure 6:**
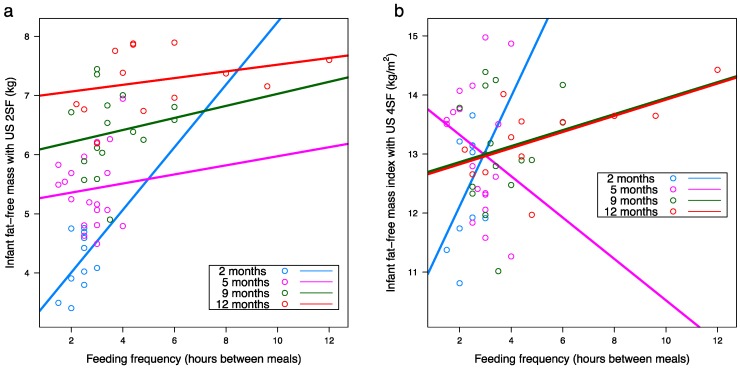
Significant positive associations between: (**a**) self-reported feeding frequency (hours between feeds) and infant fat-free mass measured with ultrasound (2 skinfolds; US 2SF) (*n* = 11, *n* = 19, *n* = 16, *n* = 13 at 2, 5, 9 and 12 months respectively); (**b**) self-reported feeding frequency and infant fat-free mass index measured with ultrasound (4 skinfolds; US 4SF) (*n* = 10, *n* = 18, *n* = 16, *n* = 12 at 2, 5, 9 and 12 months respectively). Lines represent linear regression, one line for each time point.

After adjusting for false discovery rate, longer intervals between feeds (self-reported FFQ) were associated with larger infant FFM (US 2SF: *p* = 0.001; US 4SF: *p* < 0.001; BIS: *p* = 0.019) and FFMI (US 2SF: *p* = 0.013; US 4SF: *p* < 0.001; BIS: *p* = 0.017) (Table A2, Figure 6). No significant associations were seen for 24-h MP FFQ (meals per 24 h).

Significant interactions between breastfeeding parameters and the month after birth were seen for infant BC characteristics (Table A2). 24-h MI and the month after birth: the slope for infant BMI changes from positive (5 months) to flat (9 months) and then negative (12 months) (*p* = 0.018) indicating that associations between 24-h MI and infant BMI weakens over the first 12 months of lactation; the slope for infant FFMI measured with US 4SF changes from flat (5 and 9 months) to negative (12 months) (*p* = 0.024) indicating that associations between 24-h MI and infant FFMI strengthens over the first 12 months of lactation. 24-h MP FFQ and the month after birth: the slope for infant FM measured with US 2 SF changes from negative (5 months) to positive (9 and 12 months) (*p* = 0.014) indicating that associations between FFQ and infant FM strengthens over the first 12 months of lactation.

After adjusting for false discovery rate, no associations were seen between changes in infant BC and either 24-h MI (*p* ≥ 0.0004) or FFQ (*p* ≥ 0.001 for self-reported FFQ, and *p* ≥ 0.004 for 24-h MP FFQ) at any practical time points (Table A3 and Table A4).

### 3.9. Maternal Body Composition and Breastfeeding Parameters

No associations were seen between maternal BC and 24-h MI and both FFQ (24-h MP and self-reported) (BMI: *p* ≥ 0.45; FFM: *p* ≥ 0.51; FFMI: *p* ≥ 0.13; FM: *p* ≥ 0.82; FMI: *p* ≥ 0.69; %FM: *p* ≥ 0.67) after accounting for the month after birth (*p* < 0.001 for all) and interaction between month after birth and maternal characteristics. 

Significant interaction was seen between maternal BMI and the month after birth (2 months: reference; 5 months: −0.03 ± 0.07; 9 months: 0.01 ± 0.07; 12 months: 0.76 ± 0.17, *p* < 0.001; month after birth: *p* < 0.001) and maternal FFMI and the month after birth (2 months: reference; 5 months: −0.13 ± 0.16; 9 months: 0.11 ± 0.16; 12 months: 1.18 ± 0.22, *p* < 0.001; month after birth: *p* < 0.001) for self-reported FFQ indicating that the association between both, BMI and FFMI, and self-reported FFQ (hours between feeds) strengthens over the first 12 months of lactation. No significant interaction with month after birth was seen for other maternal characteristics.

After adjusting for the false discovery rate, no associations were seen between decrease in maternal BMI, FFM, FFMI, FM, %FM and FMI and either 24-h MI (*p* ≥ 0.009) or FFQ (self-reported FFQ: *p* ≥ 0.014; 24-h MP FFQ: *p* ≥ 0.068) at any practical time points (Table A5).

## 4. Discussion

**Figure 7:**
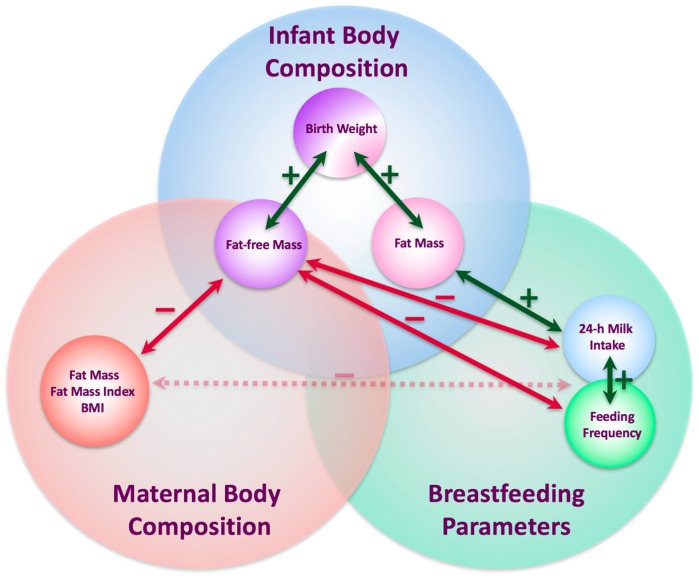
Interconnecting pathways of lactocrine programming of the infant as researched. Green arrows indicate positive associations between measured parameters; red arrows indicate negative associations; red dotted arrow indicates significant interaction terms (negative). BMI—body mass index.

The life period spanning from pre-conception to early life is a critical period when appetite control and BC are programmed and is the greatest window of opportunity for intervention to significantly improve infant outcome. This period is influenced by the maternal factors and early nutrition [5] and breastfeeding can have long-term beneficial health effects at both the individual and population levels [4]. Furthermore, longer duration of breastfeeding is shown to reduce risk for rapid growth patterns in early childhood [59] and attenuate the adverse effects of BW and early weight gain on infant FM gain [27], suggesting dose-dependent effect of breastfeeding on development of infant BC, but the mechanisms of this effect are not fully understood. Our study expands previous research, identifies specific risk factors and critical periods and sheds new light on the mechanisms by which breastfeeding influences infant BC. FFQ and 24-h MI are implicated in development of infant FM while maternal BC is associated with infant FFM, all of these emphasizing the critical role of breastfeeding in programming growth in the first 12 months of life (Figure 7). 

Milk intake is a major driver of infant growth and here we show a link between infant breastfeeding behavior, as in FFQ, where more frequent feeders consumed more milk and subsequently had greater adiposity (FM, %FM, FMI) and less lean mass (FFM, FFMI) over the first 12 months of life. This supports a study, in which 24-h MI had no associations with infant weight, but was positively associated with both weight-for-length and weight-for-age [60], suggesting infant BC rather than weight drives this relationship [61]. The lack of association between FFQ and infant 24h-MI in previous studies of the exclusive breastfeeding period is likely due to no consensus on definitions of a breastfeed or meal and non-compliance to demand feeding [31,62]. One cross-sectional study has reported a positive association between FFQ and 24-h MI, however, not all mothers exclusively breastfed for 6 months and study did not account for FFQ in early life [63], which is known to reduce in established lactation [64]. In our study, the association between FFQ and infant adiposity strengthened with duration of lactation, similar to the study, which found that the later breastfeeding was discontinued the more infant %FM was observed at 6 months [65], further highlighting the importance of breastfeeding in the weaning period during the gradual introduction of food. 

It is not fully understood what influences infant FFQ, which generally declines with the duration of lactation [62,64]. We have previously reported that smaller, shorter and leaner (less %FM), but not younger infants fed more frequently in a cohort of 2 and 5 month-old fully breastfed infants [32] and we have now extended this relationship to FFM, although it is not clear which comes first: are smaller infants in greater demand for nutrients, or is higher FFQ/MI provide more HM components that may regulate/slow down the growth? The results of this study were not uniform between self-reported FFQ and 24-h MP FFQ. This could be explained by the fact that both methods of measuring FFQ have some limitations: self-reported FFQ was shown to be biased towards reporting higher numbers of feeds in frequent feeders compared with 24-h MP FFQ, which itself is limited to one measure at the time point of data collection. Nevertheless, the associations between infant BC and both FFQ and 24-h MI indicate that, with more comprehensive investigation, these provide a window for adjustment of infant growth rate, during both, exclusive breastfeeding period and specifically from 9 to 12 months, where HM could potentially make a greater contribution to optimal infant growth rate and adiposity (Figure 1).

Previous studies based on maternal BMI [14,15] or BC measured during pregnancy [23,24,25,26,66] reported a positive relationship with infant BW. Our study showed no relationship of BW with either maternal BMI or BC, although none of our mothers were categorised as underweight and only 15% were obese, and correction for multiple comparisons eliminated statistical significance. However, we did find that increased maternal adiposity was related to lower infant FFM over the course of the first year of life whereas only one previous cross-sectional study has found this at 5 to 35 days of life [67]. Therefore, this sustained relationship suggests maintenance of healthy maternal pre-pregnancy and possibly lactational adiposity is beneficial for the development of infant lean body mass. Optimal lean body mass is desirable, since development of obesity is associated with increased FFM [68] and compositional changes of the lean tissue [69]. Further, we show in breastfed infants increased BW is associated with increased FFM throughout the first year of life, which further emphasizes the necessity for maternal BC to remain within the normal range. Interestingly, FFM at birth has also been shown to explain most of the variability in BW [70,71,72], although this finding is not consistent [19,73].

Previous studies also have linked maternal pre-pregnancy BMI or gestational weight gain to increased infant FM or %FM either very early in life (birth to 4 months) or later in childhood (2–11 years) [19,20,67,74,75]. Mechanistic studies have not been attempted to understand how breastfeeding in early life is related to development of infant adiposity. Similar to one recent longitudinal study that showed no relationship between pre-pregnancy BMI and infant postpartum %FM during first 6 months [76], we found no relationship of estimated pre-pregnancy/current maternal BC and infant FM, leading us to speculate that breastfeeding may modulate infant FM development, contributing in part to protection against obesity. Further, other studies have not measured breastfeeding parameters and have included formula-fed infants, this combined with the historic cohorts including mothers with BMI significantly lower than current cohorts [77] may have influenced their findings. 

BW is not a reliable predictor of newborn adiposity [78]. We found no association between BW and infant FM during the first 12 months of life, with the exception of a positive association at 5 months, when measured with BIS only. Similarly, Chomtho et al. have reported positive association between BW and infant FM measured with stable isotope at 3 months [73]. The emergence of positive associations of BW with infant FM only at 3 months [73] and 5 months (our study) in the first year of life might be explained by relative proximity of the measures to the peaking of infant adiposity at around 6–7 months [38,79] and a reduction in the adverse effect of BW and early infant weight gain on FM later in infancy with increased duration of breastfeeding [27,80]. Our findings therefore point to maternal BC being implicated in lean tissue development rather than fat accrual in the infant post birth, while breastfeeding parameters appear to be involved in development of adipose tissue. 

Higher FM in infants is usually considered as a negative result [81], however some studies observed %FM to be consistently higher due to lower FFM in breastfed infants compared to formula-fed [82] which may be related to the neurodevelopmental and cognitive differences between these two groups [83]. Curiously, duration of breastfeeding has been found to associate positively with infant subcutaneous but not visceral fat [76], indicating that breastfeeding may ensure a beneficial adipose phenotype, associated with a reduced risk of NCD and obesity [76,84]. It will be necessary to study the development of visceral fat in breastfed infants over time, since it cannot be extrapolated to %FM [76].

Infant BC also influenced by the infant sex. In our recent cross-sectional study of 2, 5, 9 and 12 months old infants we showed that %FM was lower in males than in females [35]. Similar to some longitudinal studies [85,86] and contrary to others [38,87,88], we have seen no difference in adiposity between sexes in this study, although, as expected, we observed that lean mass in males was greater (FFM and FFMI). Larger sample sizes will allow for more robust findings with respect to sex and BC of breastfeeding infants. 

Given the evidence confirming that obese mothers experience greater physical difficulties in breastfeeding as well as being at higher risk of not producing adequate volumes of milk [60,61,89,90,91], one might expect maternal BC to influence breastfeeding parameters. Our study, however, did not find any associations between maternal BC or indicators of milk production (24-h MI) and breastfeeding behaviour (FFQ). This may be because all of our mothers produced enough milk for their infants [31] and that only 15% of our mothers were obese. Furthermore, infants in our study displayed appropriate patterns of growth according to WHO growth standards [92], with 18 out of 20 infants residing between 15th and 97th weight-for-age centiles over the first year of life, and only three infants crossing two major centiles in downward and one in upward trend from birth to 12 months. Before correction for multiple comparisons however, we found that the reduction in maternal adiposity was associated with higher 24-h MI in the later stages of lactation, these findings are further supported by significant interaction between maternal BMI and the month after birth, which indicated the strengthening of the association between BMI and FFQ at the later stages of lactation. It is biologically plausible that higher FFQ and MI at the later stages of lactation/during weaning may contribute to greater reduction in maternal adiposity due to the energetic demand of lactation. Indeed, exclusive breastfeeding promotes greater maternal %FM loss than mixed feeding during the early postpartum period [93], and at the later stages of lactation [56,94], with more frequent feeding associating with greater fat reduction at 6 months postpartum [94]. One must be cautious, as FFQ is also related to 24-h MI. These findings support the limited data that suggests that duration of lactation is associated with protection against incidence of obesity, CVD, type 2 diabetes and prevalence of the metabolic syndrome [95], enabled by the mobilization of the stored fat and persisting beyond weaning.

The strength of this proof-of-concept study is the wide variation of adiposity levels among the mothers and that measurements were performed on breastfeeding dyads feeding on demand over a wide period of lactation, from 2 to 12 months. The limitations are the small number of 24-h MP at the later stages of lactation and the modest number of participants as a result of time constraints associated with multiple measurement time points. Our population was predominantly Caucasian term healthy fully breastfed singletons from mothers of higher social-economic status therefore, the results may not be applicable to dyads from other backgrounds. Further analysis including a holistic approach is required to understand multiple levels of breastfeeding programing and regulatory effects. Elucidation of the effect of maternal BC on infant BC via both composition and the quantity of HM will help to understand the intergenerational nature of obesity allowing for the possibility of interventions. These interventions would include maintaining/achieving healthy maternal BC pre-conception and during pregnancy and lactation, as well as supporting breastfeeding from 9 to 12 months and beyond, as outlined in the NHMRC [96] and WHO infant feeding guidelines [97], to improve the outcomes for breastfeeding dyads. An example of an ‘intervention’ is Norway’s promotion of breastfeeding (and extended breastfeeding), which is deployed by overweight/obese mothers as a weight loss strategy, since Norwegian women with greater pre-pregnancy weight concerns are more likely to initiate breastfeeding and breastfeed for longer [98]. 

## 5. Conclusions

This study found that infant BC was associated with both maternal adiposity and breastfeeding patterns over the first 12 months of lactation. These results confirm that the first year of life is a critical window of infant developmental programming that has the potential for intervention to improve outcomes for the infant, and emphasise the importance of including quantitative measures in order to elucidate the mechanisms by which breastfeeding affects infant BC (Figure 1).

## Figures and Tables

**Table 1:** Participant anthropometric and breastfeeding characteristics.

Characteristic	2 Months ^a^	5 Months ^b^	9 Months ^c^	12 Months ^d^
	Mean ± SD	Mean ± SD	Mean ± SD	Mean ± SD
	(Range)	(Range)	(Range)	(Range)
**Mothers**				
Weight (kg)	78.8 ± 19.3	70.1 ± 17.8	63.0 ± 10.0	64.2 ± 17.3
	(57.5–116.2)	(53.7–115.3)	(50.4–121.9)	(51.4–121.9)
BMI (kg/m^2^)	27.2 ± 5.5	24.8 ± 5.0	22.7 ± 3.9	23.9 ± 5.9
	(20.4–35.5)	(19.0–35.2)	(17.9–37.2)	(18.2–37.2)
Fat-free Mass ^e^ (kg)	49.5 ± 8.2	45.4 ± 6.6	44.1 ± 4.1	45.4 ± 6.7
	(38.2–66.2)	(37.4–60.9)	(35.1–68.5)	(35.9–67.7)
Fat Mass ^e^ (kg)	29.3 ± 11.8	24.6 ± 12.0	18.9 ± 7.4	18.8 ± 11.0
	(15.2–50.0)	(13.9–54.4)	(11.4–53.4)	(10.0–54.3)
Fat Mass ^e^ (%)	36.0 ± 6.4	33.8 ± 7.0	29.2 ± 6.7	27.7 ± 7.9
	(25.7–44.7)	(23.2–47.2)	(20.0–44.3)	(19.4–44.5)
FFMI ^e^ (kg/m^2^)	16.8 ± 2.1	16.2 ± 1.9	16.1 ± 2.3	16.4 ± 2.4
	(13.7–20.2)	(13.2–20.0)	(12.4–20.9)	(12.7–20.7)
FMI ^e^ (kg/m^2^)	9.5 ± 3.3	8.4 ± 3.3	7.9 ± 3.5	7.5 ± 3.5
	(6.2–15.3)	(4.8–16.6)	(3.9–16.3)	(3.6–16.6)
**Infants**				
Sex (M/F)	9M/6F	10M/10F	10M/9F	9M/9F
Age (months)	2.04 ± 0.14	5.16 ± 0.22	9.22 ± 0.27	12.26 ± 0.28
	(1.87–2.33)	(4.77–5.47)	(8.83–9.77)	(11.63–12.67)
Length (cm)	58.1 ± 1.9	64.8 ± 2.3	71.7 ± 1.9	73.6 ± 3.2
	(54.2–60.0)	(60.5–69.5)	(66.0–74.0)	(69.0–78.5)
Weight (kg)	5.630 ± 0.660	7.431 ± 1.134	8.836 ± 0.975	9.650 ± 0.618
	(4.420–7.400)	(5.808–9.510)	(6.675–10.095)	(7.165–11.085)
BMI (kg/m^2^)	16.6 ± 1.2	17.6 ± 1.9	17.7 ± 1.7	17.8 ± 0.9
	(14.5–18.1)	(14.9–20.4)	(14.2–20.2)	(13.7–19.2)
Head circumference (cm)	39.7 ± 1.6	42.1 ± 1.5	45.6 ± 1.7	46.6 ± 1.7
	(37.0–42.0)	(40.0–45.9)	(43.0–48.5)	(44.2–49.5)
**Breastfeeding characteristics**				
24-h milk intake (g)	n/a ^f^	818.8 ± 204.9	502.3 ± 157.8	445.5 ± 200.4
		(498–1185)	(300–775)	(255–795)
24-h feeding frequency (MP)	n/a ^f^	8.1 ± 1.4	5.4 ± 1.2	4.4 ± 1.9
		(6–11)	(4–7)	(2–8)
Feeding frequency (SR)	2.3 ± 0.4 ^g^	2.8 ± 0.8	3.7 ± 1.2	5.4 ± 2.9
	(1.5–3.0)	(1.5–4.0)	(2.0–6.0)	(2.2–12.0)

Data are mean ± SD and ranges. ^a^ *n* = 15; ^b^ *n* = 20; ^c^ *n* = 19; ^d^ *n* = 18. ^e^ Maternal body composition as measured with bioelectrical impedance spectroscopy. ^f^ Milk intake and feeding frequency as meals per 24-h was determined from 24-h milk production (MP) measured between 2 and 5 months (presented at 5 months here, *n* = 17) and within 2 weeks of 9 (*n* = 8) and 12 months (*n* = 9 for feeding frequency, *n* = 8 for milk intake). ^g^ Maternal self-report (SR) of feeding frequency at the time of the visit as a typical time between feeds (e.g., each 2 h) (*n* = 11, *n* = 19, *n* = 17, *n* = 13 at 2, 5, 9 and 12 months respectively). BMI—body mass index; FFMI—fat-free mass index; FMI—fat mass index, n/a—not applicable.

## Supplementary Materials

The following are available online at www.mdpi.com/2072-6643/10/1/45/s1, Table S1: Body composition of breastfed infants aged 2 to 12 months calculated with ultrasound skinfolds and bioelectrical impedance equations, Table S2: Significant differences by lactation duration within measured maternal and breastfeeding characteristics, Table S3: Significant differences by age within measured infant characteristics.

## Appendix A

**Table A1:** Significant associations between infant body composition and maternal body composition.

Predictor	2 Months		5 Months		9 Months		12 Months		*p*-Value		
	Intercept (SE)	Slope (SE)	Intercept (SE)	Slope (SE)	Intercept (SE)	Slope (SE)	Intercept (SE)	Slope (SE)	Predictor	Infant Age (Months)	Interaction
**Infant fat-free mass with ultrasound 2 skinfolds (kg)**											
Maternal BMI ^d^ (kg/m^2^)	5.62 (0.79) ^a^	−0.06 (0.03)	6.81 (0.69)	−0.06 (0.03)	7.78 (0.61)	−0.06 (0.03)	8.55 (0.60)	−0.06 (0.03)	**0.007** ^b^	**<0.001** ^c^	0.99 ^c^
Maternal FM ^d^ (%)	5.30 (0.84)	−0.03 (0.02)	6.22 (0.63)	−0.03 (0.02)	7.87 (0.57)	−0.05 (0.02)	8.68 (0.55)	−0.05 (0.02)	**0.004**	**<0.001**	0.40
Maternal FM (kg)	4.79 (0.40)	−0.03 (0.02)	5.81 (0.32)	−0.02 (0.01)	6.99 (0.31)	−0.03 (0.01)	7.72 (0.29)	−0.03 (0.01)	0.018	**<0.001**	0.62
Maternal FMI ^d^ (kg/m^2^)	4.91 (0.43)	−0.09 (0.05)	5.98 (0.35)	−0.07 (0.04)	7.15 (0.32)	−0.09 (0.04)	7.87 (0.31)	−0.11 (0.04)	**0.005**	**<0.001**	0.72
**Infant fat-free mass with ultrasound 4 skinfolds (kg)**											
Maternal BMI	5.90 (0.84)	−0.07 (0.03)	7.05 (0.74)	−0.07 (0.03)	7.82 (0.66)	−0.06 (0.03)	8.84 (0.65)	−0.07 (0.03)	**0.010**	**<0.001**	0.93
Maternal FM (%)	5.01 (0.88)	−0.03 (0.03)	6.06 (0.67)	−0.02 (0.02)	7.75 (0.63)	−0.04 (0.02)	8.74 (0.60)	−0.05 (0.02)	0.024	**<0.001**	0.29
Maternal FM (kg)	4.82 (0.43)	−0.03 (0.02)	5.86 (0.35)	−0.02 (0.01)	7.01 (0.33)	−0.02 (0.01)	7.90 (0.32)	−0.03 (0.01)	0.033	**<0.001**	0.54
Maternal FMI ^d^ (kg/m^2^)	4.93 (0.46)	−0.09 (0.05)	6.03 (0.38)	−0.07 (0.04)	7.17 (0.35)	−0.09 (0.04)	8.05 (0.33)	−0.11 (0.04)	**0.011**	**<0.001**	0.71
**Infant fat-free mass with bioelectrical impedance spectroscopy (kg)**											
Maternal BMI	5.76 (0.89)	−0.06 (0.03)	6.54 (0.76)	−0.05 (0.03)	7.85 (0.68)	−0.06 (0.03)	8.32 (0.68)	−0.05 (0.03)	0.030	**<0.001**	0.99
Maternal FMI ^d^ (kg/m^2^)	4.93 (0.48)	−0.07 (0.05)	5.73 (0.39)	−0.05 (0.04)	7.16 (0.36)	−0.09 (0.04)	7.72 (0.36)	−0.08 (0.04)	0.037	**<0.001**	0.79
**Infant fat-free mass index with ultrasound 2 skinfolds (kg/m^2^)**											
Maternal FM ^d^ (%)	13.90 (1.56)	−0.04 (0.05)	14.10 (1.14)	−0.04 (0.03)	15.10 (1.04)	−0.07 (0.03)	14.50 (1.00)	−0.06 (0.03)	0.038	0.12	0.85

^a^ Data are parameter estimate ± SE; effects of predictors taken from linear mixed effects models that accounted for infant age and an interaction between infant age and predictor. ^b^ Results are presented only for predictors with *p* < 0.05; after the false discovery rate adjustment, the predictor *p*-values were considered to be significant at <0.018 for infant fat-free mass measures (indicated by the bold text) and at <0.038 for fat-free mass index (none are significant). ^c^ Significant results for month after birth and the interaction between predictor and month after birth (*p* < 0.05, indicated by the bold text). ^d^ BMI—body mass index; FM—fat mass; FMI—fat mass index; %FM—percentage fat mass.

**Table A2:** Significant associations between infant body composition and feeding characteristics.

Predictor	2 Months		5 Months		9 Months		12 Months		*p*-Value		
	Intercept (SE)	Slope (SE)	Intercept (SE)	Slope (SE)	Intercept (SE)	Slope (SE)	Intercept (SE)	Slope (SE)	Predictor	Infant Age (Months)	Interaction
**Infant body mass index (kg/m^2^)**											
24-h milk intake (g) ^c^	n/a ^c^	n/a ^c^	15.20 (1.16) ^a^	0.003 (0.001)	17.7 (1.25)	0.001 (0.002)	18.90 (0.90)	−0.003 (0.002)	0.40 ^b^	0.61	**0.018** ^b^
**Infant fat mass with ultrasound 2 skinfolds (%)**											
24-h milk intake (g)	n/a	n/a	17.20 (3.45) ^a^	0.01 (0.004)	29.5 (4.72)	−0.005 (0.01)	21.50 (3.03)	0.01 (0.006)	**0.008**	0.30	0.20
**Infant fat mass with ultrasound 4 skinfolds (%)**											
Feeding frequency SR ^d^	18.40 (5.71)	3.06 (2.42)	25.40 (2.91)	0.32 (1.01)	30.50 (2.73)	−1.21 (1.01)	27.00 (2.01)	−0.64 (0.32)	0.040	0.34	0.26
Feeding frequency MP	n/a	n/a	24.30 (5.49)	0.29 (0.66)	18.9 (6.50)	1.53 (1.17)	16.60 (3.17)	1.52 (0.69)	0.029	0.16	0.38
24-h milk intake (g)	n/a	n/a	15.20 (3.30)	0.01 (0.004)	28.2 (4.46)	−0.002 (0.01)	18.90 (2.87)	0.01 (0.006)	**<0.001**	**0.043**	0.20
**Infant fat mass with ultrasound 2 skinfolds (kg)**											
24-h milk intake (g)	n/a	n/a	0.93 (0.37)	0.001 (0.0004)	2.53 (0.43)	−0.0002 (0.001)	2.10 (0.28)	0.001 (0.001)	**0.004**	**<0.001**	0.18
Feeding frequency MP	n/a	n/a	2.89 (0.57)	−0.11 (0.07)	2.00 (0.56)	0.08 (0.10)	1.94 (0.30)	0.12 (0.06)	0.63	**0.036**	**0.014**
**Infant fat mass with ultrasound 4 skinfolds (kg)**											
24-h milk intake (g)	n/a	n/a	0.81 (0.37)	0.001 (0.0004)	2.37 (0.45)	0.0001 (0.001)	1.88 (0.29)	0.001 (0.001)	**0.002**	**<0.001**	0.28
**Infant fat mass with bioelectrical impedance spectroscopy (kg)**											
24-h milk intake (g)	n/a	n/a	1.20 (0.44)	0.001 (0.001)	1.79 (0.57)	0.001 (0.001)	2.22 (0.41)	0.0004 (0.001)	0.045	**0.030**	0.65
**Infant fat mass index with ultrasound 2 skinfolds (kg/m^2^)**											
24-h milk intake (g)	n/a	n/a	2.17 (0.79)	0.003 (0.001)	4.88 (1.0)	0.0001 (0.002)	3.57 (0.72)	0.002 (0.001)	**0.001**	0.060	0.25
Feeding frequency MP	n/a	n/a	4.39 (1.28)	0.04 (0.16)	2.90 (1.57)	0.35 (0.28)	2.55 (0.82)	0.37 (0.17)	0.046	0.42	0.32
**Infant fat mass index with ultrasound 4 skinfolds (kg/m^2^)**											
Feeding frequency MP	n/a	n/a	3.81 (1.34)	0.12 (0.16)	2.82 (1.58)	0.39 (0.28)	2.36 (0.82)	0.36 (0.17)	0.025	0.14	0.52
24-h milk intake (g)	n/a	n/a	1.78 (0.77)	0.004 (0.001)	4.76 (1.03)	0.0003 (0.002)	2.91 (0.74)	0.002 (0.002)	**<0.001**	**0.022**	0.23
**Infant fat mas index with bioelectrical impedance spectroscopy (kg/m^2^)**											
24-h milk intake (g)	n/a	n/a	2.71 (0.92)	0.003 (0.001)	3.56 (1.18)	0.001 (0.002)	4.37 (0.85)	0.00003 (0.002)	0.029	0.75	0.31
**Infant fat-free mass with ultrasound 2 skinfolds (kg)**											
Feeding frequency SR	4.41 (0.61)	−0.09 (0.25)	4.94 (0.32)	0.17 (0.11)	5.82 (0.31)	0.16 (0.11)	6.58 (0.24)	0.10 (0.03)	**0.001**	**<0.001**	0.67
**Infant fat-free mass with ultrasound 4 skinfolds (kg)**											
Feeding frequency SR	4.14 (0.51)	0.04 (0.21)	5.19 (0.28)	0.09 (0.09)	5.56 (0.27)	0.24 (0.09)	6.58 (0.22)	0.13 (0.03)	**<0.001**	**<0.001**	0.25
**Infant fat-free mass with bioelectrical impedance spectroscopy (kg)**											
Feeding frequency SR	4.37 (0.75)	−0.001 (0.32)	5.14 (0.41)	0.06 (0.14)	5.69 (0.37)	0.22 (0.14)	6.74 (0.29)	0.08 (0.04)	**0.019**	**<0.001**	0.48
**Infant fat-free mass index with ultrasound 2 skinfolds (kg/m^2^)**											
Feeding frequency SR	12.10 (1.26)	0.19 (0.53)	13.10 (0.67)	−0.07 (0.23)	12.80 (0.61)	0.08 (0.23)	11.90 (0.46)	0.19 (0.07)	**0.013**	0.20	0.68
**Infant fat-free mass index with ultrasound 4 skinfolds (kg/m^2^)**											
Feeding frequency SR	11.50 (1.01)	0.44 (0.43)	13.40 (0.57)	−0.13 (0.19)	12.20 (0.51)	0.26 (0.19)	11.90 (0.39)	0.25 (0.06)	**<0.001**	**0.031**	0.25
24-h milk intake (g)	n/a	n/a	13.60 (0.80)	−0.001 (0.001)	13.30 (0.82)	−0.001 (0.002)	15.40 (0.60)	−0.004 (0.001)	**0.015**	0.057	**0.024**
**Infant fat-free mass index with bioelectrical impedance spectroscopy (kg/m^2^)**											
Feeding frequency SR	12.20 (1.15)	0.30 (0.49)	13.10 (0.62)	−0.15 (0.21)	12.40 (0.57)	0.21 (0.21)	12.20 (0.44)	0.15 (0.06)	**0.017**	0.16	0.49

^a^ Data are parameter estimate ± SE; effects of predictors taken from linear mixed effects models that accounted for infant age and an interaction between infant age and predictor. ^b^ Results are presented only for interactions (indicated by the bold text) or predictors with *p* < 0.05; after the false discovery rate adjustment, the predictor *p*-values were considered to be significant at <0.029 for 24-h milk intake, at <0.040 for self-reported feeding frequency (indicated by the bold text) and at <0.05 for 24-h milk production feeding frequency (none are significant). ^c^ 24-h milk intake and feeding frequency as meals per 24-h (MP) were measured between 2 and 5 months (*n* = 17; presented here at 5 months) and within 2 weeks of 9 (*n* = 8) and 12 months (*n* = 9 for feeding frequency, *n* = 8 for milk intake). ^d^ Feeding frequency was self-reported by mothers (SR) at the time of the visit as a typical time between feeds (e.g., each 2 h) (*n* = 11, *n* = 19, *n* = 17, *n* = 13 at 2, 5, 9 and 12 months respectively). n/a—not applicable.

**Table A3:** Associations between 24-h milk intake at given time points and infant body composition changes between time points.

Changes in Infant Characteristic	Months after Birth					
	5 and 2	9 and 2	12 and 2	9 and 5	12 and 5	12 and 9
**24-h milk intake between 2 and 5 months ^e^**						
BMI ^d^ (kg/m^2^)	**0.014 ^a,c^** **0.003 ± 0.001 ^b^**	0.54 0.001 ± 0.002	0.94 0.0002 ± 0.002	0.55 −0.001 ± 0.001	0.29 −0.002 ± 0.002	0.35 −0.001 ± 0.001
Fat-free mass US 2SF ^d^ (kg)	0.65 −0.004 ± 0.001	**0.013** **0.002 ± 0.001**	0.10 0.002 ± 0.001	0.56 0.004 ± 0.001	0.56 0.004 ± 0.001	0.88 0.0 ± 0.0
Fat-free mass US 4SF ^d^ (kg)	0.35 0.001 ± 0.001	**0.004** **0.003 ± 0.001**	**0.007** **0.003 ± 0.001**	0.38 0.001 ± 0.001	0.29 0.001 ± 0.001	0.26 0.0004 ± 0.003
Fat-free mass BIS ^d^ (kg)	0.096 0.001 ± 0.0003	0.14 0.002 ± 0.001	**0.009** **0.003 ± 0.001**	0.46 0.001 ± 0.001	0.20 0.001 ± 0.001	0.23 0.001 ± 0.001
Fat-free mass index US 4SF (kg/m^2^)	0.068 0.003 ± 0.001	0.092 0.003 ± 0.001	**0.035** **0.005 ± 0.002**	0.63 0.001 ± 0.001	0.84 0.0003 ± 0.001	0.91 0.0001 ± 0.001
Fat-free mass index BIS (kg/m^2^)	**0.031** **0.002 ± 0.001**	0.47 0.001 ± 0.002	0.37 0.002 ± 0.002	0.68 0.001 ± 0.001	0.92 0.0001 ± 0.001	0.68 0.001 ± 0.001
Fat mass US 2SF (kg)	**0.037** **0.002 ± 0.001**	0.46 0.001 ± 0.001	0.47 0.001 ± 0.001	0.77 −0.0002 ± 0.001	0.60 −0.0003 ± 0.001	0.97 0.0 ± 0.0
Fat mass index US 2SF (kg/m^2^)	**0.033** **0.004 ± 0.002**	0.98 0.0 ± 0.0	0.87 −0.0004 ± 0.002	0.50 −0.001 ± 0.001	0.25 −0.002 ± 0.001	0.39 −0.001 ± 0.001
Fat mass index US 4SF (kg/m^2^)	0.47 0.001 ± 0.001	0.23 −0.003 ± 0.002	0.19 −0.004 ± 0.003	0.38 −0.001 ± 0.001	0.074 −0.002 ± 0.001	**0.047** **−0.001 ± 0.001**
Fat mass index BIS (kg/m^2^)	0.065 0.002 ± 0.001	0.97 0.0 ± 0.0	0.46 −0.001 ± 0.001	0.37 −0.001 ± 0.001	**0.034** **−0.003 ± 0.001**	0.21 −0.002 ± 0.001
**24-h milk intake at 12 months ^e^**						
Fat-free mass index US 2SF (kg/m^2^)	n/a ^f^	n/a ^f^	0.14 −0.005 ± 0.002	n/a ^f^	**0.003** **−0.006 ± 0.001**	0.064 −0.003 ± 0.001
Fat-free mass index US 4SF (kg/m^2^)	n/a	n/a	0.31 −0.005 ± 0.004	n/a	**0.0004** **−0.005 ± 0.0004**	0.18 −0.003 ± 0.002
Fat mass US 2SF (kg)	n/a	n/a	**0.023** **0.002 ± 0.001**	n/a	0.17 0.001 ± 0.001	0.41 0.0003 ± 0.0004
Fat mass US 2SF (%)	n/a	n/a	**0.049** **0.029 ± 0.009**	n/a	0.11 0.013 ± 0.007	0.60 0.003 ± 0.006

^a^ *p*-values, ^b^ parameter estimates and standard errors of estimate for significant associations between feeding frequency at given time points and the changes in measured variables between different months after birth. ^c^ Results are presented only for variables with at least one significant raw *p*-value (*p* < 0.05, indicated by the bold text); after the false discovery rate adjustment, the predictor *p*-values were considered to be significant at <0.0004 for 24-h milk intake (none are significant). ^d^ BIS—bioimpedance spectroscopy; BMI—body mass index; US 2SF—ultrasound 2-skinfolds; US 4SF—ultrasound 4-skinfolds. ^e^ 24-h milk intake was measured at 24-h milk production between 2 and 5 months (*n* = 17) and within 2 weeks of 9 (*n* = 8) and 12 months (*n* = 8). ^f^ Results are not presented for impractical combinations, n/a—not applicable.

**Table A4:** Associations between feeding frequency at given time points and infant body composition changes between time points.

Changes in Infant Characteristic	Months after Birth					
	5 and 2	9 and 2	12 and 2	9 and 5	12 and 5	12 and 9
**Self-reported feeding frequency at 2 months ^e^**						
BMI ^d^ (kg/m^2^)	0.83 ^a^ 0.14 ± 064 ^b^	0.067 −1.95 ± 0.92	0.42 −0.99 ± 1.15	**0.013 ^c^** **−2.09 ± 0.66**	0.25 −1.13 ± 0.87	**0.027** **1.03 ± 0.37**
Fat mass US 4SF ^d^ (%)	0.55 1.34 ± 2.14	0.058 −7.19 ± 3.25	0.25 −4.67 ± 3.79	**0.027** **−9.11 ± 3.37**	0.13 −5.80 ± 3.40	0.28 3.20 ± 2.74
Fat mass US 4SF (kg)	0.37 0.14 ± 0.15	0.12 −0.59 ± 0.34	0.23 −0.44 ± 0.33	**0.028** **−0.76 ± 0.28**	0.076 −0.56 ± 0.28	0.42 0.17 ± 0.20
Fat mass index US 4SF (kg/m^2^)	0.28 0.40 ± 0.35	**0.039** **−1.73 ± 0.70**	0.27 −0.97 ± 0.81	**0.009** **−2.13 ± 0.62**	0.094 −1.35 ± 0.70	0.14 0.83 ± 0.49
**Self-reported feeding frequency at 9 months ^e^**						
Fat-free mass US 4SF (kg)	n/a ^g^	0.055 0.21 ± 0.10	0.25 0.21 ± 0.17	**0.003** **0.25 ± 0.07**	0.13 0.18 ± 0.11	0.93 −0.01 ± 0.09
Fat-free mass index US 4SF (kg/m^2^)	n/a	0.10 0.29 ± 0.16	0.37 0.29 ± 0.31	**0.014** **0.36 ± 0.13**	**0.025** **0.48 ± 0.19**	0.49 0.13 ± 0.18
**Self-reported feeding frequency at 12 months ^e^**						
BMI (kg/m^2^)	n/a ^g^	n/a ^g^	0.45 0.15 ± 0.18	n/a ^g^	0.12 0.21 ± 0.12	**0.044** **0.14 ± 0.06**
Fat-free mass US 2SF ^d^ (kg)	n/a	n/a	**0.031** **0.13 ± 0.04**	n/a	**0.022** **0.12 ± 0.05**	0.43 0.03 ± 0.03
Fat-free mass US 4SF (kg)	n/a	n/a	**0.047** **0.15 ± 0.06**	n/a	**0.001** **0.15 ± 0.03**	0.21 0.04 ± 0.03
Fat-free mass index US 2SF (kg/m^2^)	n/a	n/a	**0.024** **0.24 ± 0.07**	n/a	**0.020** **0.24 ± 0.09**	0.052 0.12 ± 0.06
Fat-free mass index US 4SF (kg/m^2^)	n/a	n/a	**0.043** **0.31 ± 0.10**	n/a	**0.003** **0.27 ± 0.07**	0.073 0.15 ± 0.08
Fat-free mass index BIS ^d^ (kg/m^2^)	n/a	n/a	0.051 0.25 ± 0.09	n/a	**0.030** **0.16 ± 0.06**	0.37 0.07 ± 0.07
Fat mass US 4SF (%)	n/a	n/a	**0.043** **−1.05 ± 0.39**	n/a	0.11 −0.67 ± 0.38	0.44 −0.26 ± 0.32
**24-h MP feeding frequency between 2 and 5 months ^f^**						
Fat-free mass index US 2SF (kg/m^2^)	**0.028** **0.45 ± 0.17**	0.16 −0.27 ± 0.17	0.52 −0.17 ± 0.25	0.13 −0.28 ± 0.17	0.29 −0.21 ± 0.19	0.64 0.06 ± 0.12
Fat-free mass index US 4SF (kg/m^2^)	**0.012** **0.45 ± 0.13**	0.51 −0.16 ± 0.23	0.80 −0.09 ± 0.36	0.065 −0.25 ± 0.12	0.63 −0.09 ± 0.18	0.46 0.11 ± 0.15
Fat mass US 2SF (%)	0.39 −1.60 ± 1.75	0.24 1.20 ± 0.94	0.48 1.34 ± 1.81	**0.012** **2.23 ± 0.78**	0.068 1.70 ± 0.86	0.21 −0.51 ± 0.38
Fat mass US 4SF (%)	0.23 −0.96 ± 0.73	0.53 0.93 ± 1.38	0.62 0.90 ± 1.73	**0.010** **2.09 ± 0.69**	0.12 1.26 ± 0.77	0.086 −0.76 ± 0.41
Fat mass US 2SF (kg)	0.62 −0.08 ± 0.15	0.23 0.14 ±0.10	0.48 0.13 ± 0.17	**0.007** **0.21 ± 0.07**	**0.033** **0.17 ± 0.07**	0.34 −0.04 ± 0.04
Fat mass US 4SF (kg)	0.81 −0.01 ± 0.05	0.24 0.14 ± 0.11	0.38 0.12 ± 0.13	**0.006** **0.21 ± 0.06**	0.054 0.14 ± 0.07	0.17 −0.05 ± 0.04
Fat mass index US 2SF (kg/m^2^)	0.64 −0.16 ± 0.32	0.40 0.20 ± 0.23	0.40 0.30 ± 0.34	**0.016** **0.44 ± 0.16**	0.11 0.33 ± 0.19	0.22 −0.11 ± 0.08
Fat mass index US 4SF (kg/m^2^)	0.81 −0.03 ± 0.13	0.53 0.19 ± 0.28	0.60 0.20 ± 0.36	**0.023** **0.42 ± 0.16**	0.17 0.26 ± 0.18	0.053 −0.16 ± 0.08
	**24-h MP feeding frequency at 9 months ^f^**					
Fat-free mass US 2SF (kg)	n/a ^g^	**0.030** **−0.28 ± 0.05**	0.33 −0.32 ± 0.28	**0.013** **−0.33 ± 0.09**	0.16 −0.33 ± 0.20	0.85 −0.03 ± 0.15
Fat-free mass index US 4SF (kg/m^2^)	n/a	0.33 −0.45 ± 0.35	0.43 −0.55 ± 0.57	0.14 −0.35 ± 0.19	**0.044** **−0.68 ± 0.23**	0.15 −0.35 ± 0.20
**24-h MP feeding frequency at 12 months ^f^**						
Fat-free mass US 2SF (kg)	n/a ^g^	n/a ^g^	**0.029** **−0.23 ± 0.06**	n/a ^g^	0.097 −0.20 ± 0.11	0.55 −0.05 ± 0.07
Fat-free mass index US 2SF (kg/m^2^)	n/a	n/a	0.070 −0.41 ± 0.12	n/a	**0.037** **−0.48 ± 0.18**	0.45 −0.13 ± 0.15
Fat mass US 2SF (%)	n/a	n/a	0.13 2.00 ± 0.97	n/a	**0.004** **2.12 ± 0.52**	0.96 0.03 ± 0.61
Fat mass US 2SF (kg)	n/a	n/a	0.090 0.15 ± 0.06	n/a	**0.009** **0.19 ± 0.05**	0.90 0.01 ± 0.04
Fat mass index US 2SF (kg/m^2^)	n/a	n/a	0.30 0.24 ± 0.17	n/a	**0.015** **0.41 ± 0.12**	0.75 −0.04 ± 0.11

^a^ *p*-values, ^b^ parameter estimates and standard errors of estimate for significant associations between feeding frequency at given time points and the changes in measured variables between different months after birth. ^c^ Results are presented only for variables with at least one significant raw *p*-value (*p* < 0.05, indicated by the bold text); after the false discovery rate adjustment, the predictor *p*-values were considered to be significant at <0.001 for self-reported feeding frequency, and <0.004 for 24-h milk production feeding frequency (none are significant). ^d^ BIS—bioimpedance spectroscopy; BMI—body mass index; MP—milk production; US 2SF—ultrasound 2-skinfolds; US 4SF—ultrasound 4-skinfolds. ^e^ Feeding frequency was self-reported by mothers at the time of the visit as a typical time between feeds (e.g., each 2 h) (*n* = 11, *n* = 19, *n* = 17, *n* = 13 at 2, 5, 9 and 12 months respectively). ^f^ Feeding frequency as meals per 24-h was measured at 24-h milk production between 2 and 5 months (presented at 5 months here, *n* = 17) and within 2 weeks of 9 (*n* = 8) and 12 months (*n* = 9). ^g^ Results are not presented for impractical combinations, n/a—not applicable.

**Table A5:** Associations between breastfeeding parameters at given time points and maternal body composition changes between time points.

Changes in Maternal Characteristic	Months after Birth					
	5 and 2	9 and 2	12 and 2	9 and 5	12 and 5	12 and 9
**24-h milk intake at 9 months ^e^**						
Fat mass BIS ^d^ (%)	n/a ^g^	0.075 ^a^ 0.04 ± 0.01 ^b^	0.33 0.01 ± 0.01	**0.042 ^c^** **−0.01 ± 0.004**	**0.013** **−0.02 ± 0.005**	0.16 −0.007 ± 0.004
Fat mass BIS (kg)	n/a	**0.026** **0.04 ± 0.01**	0.28 0.01 ± 0.01	0.10 −0.006 ± 0.003	**0.011** **−0.01 ± 0.003**	0.16 −0.005 ± 0.003
Fat mass index BIS (kg/m^2^)	n/a	**0.020** **0.02 ± 0.002**	0.26 0.003 ± 0.002	0.11 −0.002 ± 0.001	**0.009** **−0.004 ± 0.001**	0.15 −0.002 ± 0.001
**Self-reported feeding frequency at 12 months ^f^**						
BMI ^d^ (kg/m^2^)	n/a ^g^	n/a ^g^	**0.025** **0.15 ± 0.05**	n/a ^g^	0.96 −0.003 ± 0.05	0.74 0.02 ± 0.05
Fat mass BIS (%)	n/a	n/a	**0.048** **0.38 ± 0.15**	n/a	0.83 0.07 ± 0.32	0.63 0.10 ± 0.20
Fat mass BIS (kg)	n/a	n/a	**0.020** **0.38 ± 0.11**	n/a	0.71 0.08 ± 0.20	0.59 0.08 ± 0.15
Fat mass index BIS (kg/m^2^)	n/a	n/a	**0.014** **0.12 ± 0.03**	n/a	0.78 0.02 ± 0.07	0.58 0.03 ± 0.05

^a^ *p*-values, ^b^ parameter estimates and standard errors of estimate for significant associations between breastfeeding parameters at given time points and the changes in measured variables between different months after birth. ^c^ Results are presented only for variables with at least one significant raw *p*-value (*p* < 0.05; indicated by the bold text; none were significant for 24-h milk production feeding frequency); after the false discovery rate adjustment, the predictor *p*-values were considered to be significant at <0.009 for 24-h milk intake and <0.014 for self-reported feeding frequency (none are significant). ^d^ BIS—bioimpedance spectroscopy; BMI—body mass index. ^e^ 24-h milk intake was measured at 24-h milk production between 2 and 5 months (*n* = 17) and within 2 weeks of 9 (*n* = 8) and 12 months (*n* = 8). ^f^ Feeding frequency was self-reported by mothers at the time of the visit as a typical time between feeds (e.g., each 2 h) (*n* = 11, *n* = 19, *n* = 17, *n* = 13 at 2, 5, 9 and 12 months respectively). ^g^ Results are not presented for impractical combinations, n/a—not applicable.
