# Supplementary material for: Relationships between Breastfeeding Patterns and Maternal and Infant Body Composition over the First 12 Months of Lactation
Source: Nutrients. 2018 Jan 5;10(1):45. doi: 10.3390/nu10010045 (PMC5793273; doi:10.3390/nu10010045)
Supplement: Supplementary file 1 [file nutrients-10-00045-s001.pdf]

Supplementary Materials: Relationships Between Breastfeeding Patterns and Maternal and Infant Body Composition Over the First 12 Months of Lactation

Zoya Gridneva, Alethea Rea, Anna R. Hepworth, Leigh C. Ward, Ching T. Lai, Peter E. Hartmann and Donna T. Geddes

Table S1. Body composition of breastfed infants aged 2 to 12 months calculated with ultrasound skinfold and bioelectrical impedance equations [a].

	2 months			5 months			9 months			12 months		
**Equations**	**Female**	**Male**	**Pooled**	**Female**	**Male**	**Pooled**	**Female**	**Male**	**Pooled**	**Female**	**Male**	**Pooled**
Ultrasound 2-skinfolds	*n* = 6	*n* = 8	*n* = 14	*n* = 10	*n* = 10	*n* = 20	*n* = 9	*n* = 9	*n* = 18	*n* = 9	*n* = 9	*n* = 18
**FFM (kg)**	3.74±0.27 [b]	4.42±0.36	4.23±0.44	5.08±0.49	5.72±0.60	5.52±0.77	6.13±0.66	6.65±0.57	6.56±0.68	6.86±0.78	7.30±0.64	7.15±0.58
**FM (kg)**	1.21±0.25	1.63±0.53	1.40±0.25	1.78±0.29	2.24±0.55	1.91±0.41	2.19±0.38	2.35±0.66	2.28±0.60	2.24±0.39	2.66±0.58	2.50±0.29
**FM (%)**	24.2±3.1	26.5±5.1	24.7±2.3	25.8±2.6	27.9±5.3	25.6±2.4	26.3±3.3	25.9±5.5	25.6±5.0	24.5±2.9	26.5±4.7	26.0±3.0
**FFMI (kg/m^2)**	11.5±0.5	12.9±0.5	12.2±0.9	12.5±1.1	13.3±1.1	12.9±1.2	12.8±1.2	13.2±0.7	13.0±1.0	12.7±1.2	12.9±0.7	12.7±1.0
**FMI (kg/m^2)**	3.7±0.6	4.3±0.6	3.9±0.6	4.4±0.7	5.2±1.2	4.8±1.1	4.6±0.9	4.7±1.3	4.6±1.1	4.1±0.8	4.7±1.0	4.5±0.9
Ultrasound 4-skinfolds	*n* = 6	*n* = 7	*n* = 13	*n* = 10	*n* = 9	*n* = 19	*n* = 9	*n* = 9	*n* = 18	*n* = 9	*n* = 9	*n* = 18
**FFM (kg)**	3.69±0.30	4.53±0.44	4.20±0.47	5.05±0.49	5.84±0.61	5.53±0.77	6.10±0.69	6.74±0.58	6.62±0.67	6.92±0.81	7.56±0.64	7.28±0.56
**FM (kg)**	1.25±0.26	1.54±0.40	1.43±0.24	1.81±0.37	2.12±0.46	1.90±0.40	2.21±0.52	2.26±0.47	2.22±0.53	2.18±0.44	2.40±0.46	2.37±0.41
**FM (%)**	25.2±3.5	25.1±3.6	25.3±2.5	26.2±3.6	26.5±3.9	25.4±2.3	26.5±5.2	24.9±3.7	24.9±4.3	23.9±3.6	24.0±3.5	24.5±3.8
**FFMI (kg/m^2)**	11.3±0.5	13.0±0.6	12.2±1.0	12.4±0.9	13.7±0.9	13.0±1.1	12.8±1.0	13.4±0.8	13.1±0.9	12.8±1.2	13.4±0.6	13.0±1.0
**FMI (kg/m^2)**	3.8±0.7	4.2±0.5	4.0±0.6	4.5±1.0	5.0±0.9	4.7±1.0	4.7±1.3	4.5±0.9	4.6±1.1	4.0±0.9	4.2±0.8	4.2±0.8
Bioimpedance spectroscopy	*n* = 6	*n* = 7	*n* = 13	*n* = 10	*n* = 10	*n* = 20	*n* = 9	*n* = 9	*n* = 18	*n* = 9	*n* = 7	*n* = 16
**FFM (kg)**	3.84±0.27	4.56±0.33	4.36±0.43	4.79±0.37	5.76±0.39	5.19±0.70	6.07±0.66	6.82±0.74	6.64±0.79	6.89±0.85	7.24±0.59	7.27±0.67
**FM (kg)**	1.10±0.22	1.30±0.26	1.27±0.26	2.07±0.40	2.19±0.48	2.24±0.50	2.25±0.53	2.18±0.52	2.20±0.60	2.21±0.46	2.62±0.57	2.38±0.32
**FM (%)**	22.1±2.3	22.0±2.5	22.4±2.6	30.0±3.0	27.3±3.2	29.9±3.2	26.9±5.0	24.2±4.9	24.8±5.4	24.3±4.1	26.4±3.9	24.7±3.4

**FFMI (kg/m²)**	11.8±0.5	13.4±0.4	12.6±1.0	11.8±0.9	13.4±0.6	12.6±1.1	12.7±1.1	13.5±1.0	13.1±1.1	12.7±1.1	13.0±0.6	12.8±0.9
**FMI (kg/m²)**	3.4±0.5	3.8±0.6	3.6±0.6	5.1±0.9	5.1±0.9	5.1±0.9	4.7±1.2	4.3±1.0	4.5±1.1	4.1±1.0	4.7±0.9	4.4±1.0

[a] Body composition (%FM - percentage fat mass; FM - fat mass; FFM - fat-free mass; FFMI – fat-free mass index; FMI – fat mass index) of breastfed infants aged 2, 5, 9 and 12 months calculated with various equations: ultrasound 2-skinfolds – Slaughter et al. [41] at all ages; ultrasound 4-skinfolds – Brook [42] at all ages; bioimpedance spectroscopy – Lingwood et al. [36] 3 months-old equation at 2 months and 4.5 months-old equation at 5 months; and Bocage [37] at 9 and 12 months. [b] Data are mean ± SD and ranges.

Table S2. Significant differences by lactation duration within measured maternal and breastfeeding characteristics [a].

Characteristic	Months after birth						
	**5 and 2**	**9 and 2**	**12 and 2**	**9 and 5**	**12 and 5**	**12 and 9**	***p* overall**
Maternal							
Weight (kg)	0.37<br>-1.10 ± 0.68 [c]	**0.027** [b]<br>**-1.98 ± 0.71**	**0.009**<br>**-2.23 ± 0.71**	0.50<br>-0.88 ± 0.62	0.27<br>-1.13 ± 0.62	0.98<br>-0.25 ± 0.64	**0.014**
BMI [f] (kg/m²)	0.35<br>-0.38 ± 0.23	**0.016**<br>**-0.71 ± 0.24**	**0.007**<br>**-0.78 ± 0.24**	0.40<br>-0.33 ± 0.21	0.25<br>-0.39 ± 0.21	0.99<br>-0.06 ± 0.22	**0.009**
Fat mass (kg)	0.99<br>-0.15 ± 0.50	0.25<br>-0.97 ± 0.52	**0.003**<br>**-1.81 ± 0.52**	0.28<br>-0.82 ± 0.46	**0.002**<br>**-1.67 ± 0.46**	0.27<br>-0.85 ± 0.47	**0.002**
Fat mass (%)	0.98<br>0.22 ± 0.57	0.62<br>-0.72 ± 0.59	**0.004**<br>**-2.03 ± 0.59**	0.28<br>-0.94 ± 0.52	**<0.001**<br>**-2.25 ± 0.52**	0.066<br>-1.31 ± 0.54	**<0.001**
FMI [f] (kg/m²)	0.99<br>-0.05 ± 0.17	0.20<br>-0.35 ± 0.18	**0.002**<br>**-0.64 ± 0.18**	0.22<br>-0.30 ± 0.16	**<0.001**<br>**-0.59 ± 0.16**	0.28<br>-0.29 ± 0.16	**0.001**
Breastfeeding							
Feeding frequency (SR) [d, f]	0.82<br>0.46 ± 0.53	**0.045**<br>**1.40 ± 0.54**	**<0.001**<br>**3.14 ± 0.58**	0.17<br>0.94 ± 0.46	**<0.001**<br>**2.69 ± 0.50**	**0.003**<br>**1.75 ± 0.51**	**<0.001**
Feeding frequency (MP) [e, f]	n/a [e]	n/a	n/a	**<0.001**<br>**-2.81 ± 0.49**	**<0.001**<br>**-3.71 ± 0.46**	0.19<br>-0.90 ± 0.52	**<0.001**
24-h milk intake (g) [e, f]	n/a [e]	n/a	n/a	**<0.001**<br>**-325 ± 64**	**<0.001**<br>**-376 ± 64**	0.73<br>-52 ± 69	**<0.001**

[a] Systematic differences in the measured variables between different months after birth were calculated using general linear hypothesis test (Tukey's all pair comparisons). [b] Bold text indicates significant difference ($p < 0.05$) between two time points. Only variables with at least one significant difference between two time points are presented. [c] Data are parameter estimate and standard error of estimate. [d] Feeding frequency was self-reported by mothers (SR) at the time of the visit as a typical time between feeds (e.g., each 2 h) ($n = 11$, $n = 19$, $n = 17$, $n = 13$ at 2, 5, 9 and 12 months respectively). [e] 24-h milk intake and feeding frequency as meals per 24-h were measured at 24-h milk production between 2 and 5 months (presented at 5 months here, $n = 17$) and within 2 weeks of 9 ($n = 8$) and 12 months ($n = 9$ for feeding frequency, $n = 8$ for milk intake). [f] BMI – body mass index; FMI – fat mass index; MP – milk production; n/a – not applicable; SR – self-reported.

Table S3. Significant differences by age within measured infant characteristics [a].

Characteristic	Months after birth						
	5 and 2	9 and 2	12 and 2	9 and 5	12 and 5	12 and 9	*p* overall
Length (cm)	**<0.001** [b]<br>**6.81 ± 0.41** [c]	**<0.001**<br>**12.36 ± 0.42**	**<0.001**<br>**16.49 ± 0.42**	**<0.001**<br>**5.55 ± 0.36**	**<0.001**<br>**9.68 ± 0.37**	**<0.001**<br>**4.13 ± 0.37**	**<0.001**
Weight (kg)	**<0.001**<br>**1.78 ± 0.13**	**<0.001**<br>**3.09 ± 0.13**	**<0.001**<br>**3.85 ± 0.13**	**<0.001**<br>**1.31 ± 0.12**	**<0.001**<br>**2.08 ± 0.12**	**<0.001**<br>**0.77 ± 0.12**	**<0.001**
Head circumference (cm)	**<0.001**<br>**3.11 ± 0.20**	**<0.001**<br>**5.71 ± 0.21**	**<0.001**<br>**6.72 ± 0.21**	**<0.001**<br>**2.60 ± 0.18**	**<0.001**<br>**3.61 ± 0.19**	**<0.001**<br>**1.01 ± 0.19**	**<0.001**
BMI [d] (kg/m²)	**<0.001**<br>**1.11 ± 0.29**	**<0.001**<br>**1.14 ± 0.30**	0.17<br>0.61 ± 0.30	1.00<br>0.03 ± 0.26	0.23<br>-0.50 ± 0.26	0.20<br>-0.52 ± 0.27	**<0.001**
Fat-free mass US 2SF [d] (kg)	**<0.001**<br>**1.21 ± 0.12**	**<0.001**<br>**2.23 ± 0.12**	**<0.001**<br>**2.86 ± 0.12**	**<0.001**<br>**1.02 ± 0.11**	**<0.001**<br>**1.65 ± 0.11**	**<0.001**<br>**0.63 ± 0.11**	**<0.001**
Fat-free mass US 4SF [d] (kg)	**<0.001**<br>**1.26 ± 0.12**	**<0.001**<br>**2.28 ± 0.12**	**<0.001**<br>**3.02 ± 0.12**	**<0.001**<br>**1.02 ± 0.11**	**<0.001**<br>**1.76 ± 0.11**	**<0.001**<br>**0.74 ± 0.11**	**<0.001**
FFMI US 4SF (kg/m²)	**0.013**<br>**0.67 ± 0.22**	**0.006**<br>**0.73 ± 0.22**	**0.015**<br>**0.68 ± 0.23**	1.00<br>0.06 ± 0.19	1.00<br>0.01 ± 0.20	1.00<br>-0.05 ± 0.19	**<0.010**
Fat-free mass BIS [d] (kg)	**<0.001**<br>**0.96 ± 0.13**	**<0.001**<br>**2.15 ± 0.13**	**<0.001**<br>**2.76 ± 0.13**	**<0.001**<br>**1.20 ± 0.11**	**<0.001**<br>**1.81 ± 0.12**	**<0.001**<br>**0.61 ± 0.12**	**<0.001**
FFMI BIS (kg/m²)	0.75<br>-0.21 ± 0.21	0.50<br>0.29 ± 0.21	1.00<br>0.03 ± 0.22	**0.032**<br>**0.50 ± 0.18**	0.61<br>0.23 ± 0.19	0.51<br>-0.27 ± 0.19	0.072
Fat mass US 2SF (kg)	**<0.001**<br>**0.57 ± 0.11**	**<0.001**<br>**0.87 ± 0.12**	**<0.001**<br>**1.00 ± 0.12**	**0.017**<br>**0.30 ± 0.10**	**<0.001**<br>**0.43 ± 0.10**	0.63<br>0.13 ± 0.11	**<0.001**
FMI [d] US 2SF (kg/m²)	**0.019**<br>**0.73 ± 0.25**	0.074<br>0.62 ± 0.26	0.55<br>0.35 ± 0.26	0.96<br>-0.11 ± 0.23	0.33<br>-0.39 ± 0.23	0.64<br>-0.28 ± 0.23	**0.028**
Fat mass US 4SF (kg)	**<0.001**<br>**0.47 ± 0.10**	**<0.001**<br>**0.79 ± 0.10**	**<0.001**<br>**0.81 ± 0.10**	**0.002**<br>**0.32 ± 0.09**	**<0.001**<br>**0.33 ± 0.09**	1.00<br>0.01 ± 0.09	**<0.001**
FMI US 4SF (kg/m²)	0.018<br>0.49 ± 0.24	0.034<br>0.41 ± 0.25	0.99<br>-0.08 ± 0.25	0.98<br>-0.09 ± 0.21	**0.045**<br>**-0.57 ± 0.22**	0.11<br>-0.49 ± 0.22	**0.030**
Fat mass BIS (kg)	**<0.001**<br>0.86 ± 0.12	**<0.001**<br>0.97 ± 0.12	**<0.001**<br>1.13 ± 0.12	0.67<br>0.11 ± 0.10	0.055<br>0.27 ± 0.11	0.49<br>0.15 ± 0.11	**<0.001**

FMI BIS (kg/m^2)	**<0.001**<br>**1.34 ± 0.25**	**0.004**<br>**0.86 ± 0.25**	0.067<br>0.64 ± 0.26	0.12<br>-0.48 ± 0.22	**0.011**<br>**-0.71 ± 0.23**	0.77<br>-0.22 ± 0.23	**<0.001**
Fat mass BIS (%)	**<0.001**<br>**6.27 ± 1.12**	**0.016**<br>**3.39 ± 1.15**	0.073<br>2.85 ± 1.18	**0.021**<br>**-2.88 ± 1.01**	**0.006**<br>**-3.42 ± 1.05**	0.96<br>-0.54 ± 1.06	**<0.001**

[a] Systematic differences in the measured variables between different months after birth were calculated using general linear hypothesis test (Tukey's all pair comparisons). [b] Bold text indicates significant difference ($p < 0.05$) between two time points. Only variables with at least one significant difference between two time points are presented. [c] Parameter estimate and standard error of estimate. [d] BIS – bioimpedance spectroscopy; BMI – body mass index; FFMI – fat-free mass index; FMI – fat mass index; US 2SF – ultrasound 2-skinfolds; US 4SF – ultrasound 4-skinfolds.
